# Livestock microbial landscape patterns: Retail poultry microbiomes significantly vary by region and season

**DOI:** 10.1016/j.fm.2021.103878

**Published:** 2022-02

**Authors:** B.J. Schofield, N.A. Andreani, C.S. Günther, G.R. Law, G. McMahon, M. Swainson, M.R. Goddard

**Affiliations:** a School of Life Sciences, University of Lincoln, Lincolnshire, United Kingdom; b School of Health and Social Care, University of Lincoln, Lincolnshire, United Kingdom; c Moy Park Ltd., Craigavon, Co. Armagh, United Kingdom; d National Centre for Food Manufacturing, University of Lincoln, Lincolnshire, United Kingdom

**Keywords:** Poultry carcass, Microbiome, 16S metabarcode sequencing, Season, Region, Community profile, PERMANOVA, Permutational multivariate analysis of variance, QIIME2, Quantitative insights into microbial ecology, UK, United Kingdom

## Abstract

Microbes play key roles in animal welfare and food safety but there is little understanding of whether microbiomes associated with livestock vary in space and time. Here we analysed the bacteria associated with the carcasses of the same breed of 28 poultry broiler flocks at different stages of processing across two climatically similar UK regions over two seasons with 16S metabarcode DNA sequencing. Numbers of taxa types did not differ by region, but did by season (P = 1.2 × 10^−19^), and numbers increased with factory processing, especially in summer. There was also a significant (P < 1 × 10^−4^) difference in the presences and abundances of taxa types by season, region and factory processing stage, and the signal for seasonal and regional differences remained highly significant on final retail products. This study therefore revealed that both season and region influence the types and abundances of taxa on retail poultry products. That poultry microbiomes differ in space and time should be considered when testing the efficacy of microbial management interventions designed to increase animal welfare and food safety: these may have differential effects on livestock depending on location and timing.

## Highlights

• Retail poultry bacterial richness differs by season but not region.
• Retail poultry bacterial types differ by season and region.
• There are differential abundances of bacteria types on retail poultry by season and region.
• We infer bacterial communities become homogenised in flocks by factory processing.

## 1 Introduction

Poultry is the second largest source of animal protein consumed by humans world-wide, and over 118 million tonnes of poultry was produced globally in 2017 (OECD/FAO, 2018). Each year human food-related illness costs the UK alone £1.8 billion (FSA, 2013) and causes 310,000 to 600,000 deaths world-wide (WHO, 2015). Research has understandably concentrated on ways to reduce this burden by identifying and reducing pathogenic organisms within the microbiomes of livestock destined for human consumption.

To date the majority of poultry microbiome studies have evaluated digestive tracts due to their importance in animal health, but since viscera are typically removed and not consumed, these studies are limited in their understanding of the microbiological safety of retail poultry products. Studies that have evaluated poultry carcass microbiomes are very limited: one recent study focussed on changes in poultry carcass microbiomes through the abattoir and found a drop in bacterial load and change in microbiome in response to an immersion chilling intervention (Handley et al., 2018). We are unaware of any studies that have evaluated the effect of environmental and various food-chain processing interventions on poultry (or any other meat) carcass microbiomes destined for human consumption.

There is increasing evidence that microbiomes associated with horticultural systems differ by region (Bokulich et al., 2014; Taylor et al., 2014), and that these may correspondingly influence the quality of agricultural products (Knight et al., 2015), but there is little to no analogous data on whether livestock microbiomes display any larger scale temporal or seasonal patterns. A difference among chicken caecal microbiomes from five regions in Tibet was noted by one study, but this observation was not formally statistically tested (Zhou et al., 2016), and caeca are not usually destined for human consumption. Faecal microbiome comparisons of broilers and egg-laying hens from four different European countries showed different antibiotic resistance gene prevalence in microbial communities, but there was no comparison of overall microbiomes (Videnska et al., 2014). To our knowledge, no studies have investigated the effect of seasonality on livestock microbiomes. Knowledge of whether any microbiome patterns exist for livestock retail products is valuable to help contextualise the effects of livestock management on food safety.

Whilst Next-Generation Sequencing (NGS) approaches have been widely applied to investigate the microbiomes of chicken and other livestock gastrointestinal tracts, the microbiology of retail poultry products has been investigated with culture-dependent techniques; however, several studies have shown the majority of species in the poultry gut are not yet culturable (Shang et al., 2018). Only recently has the food sector started to move towards the application of culture-independent techniques to investigate the microbiomes of food products at different stages of the production process (Feye et al., 2020; Ricke et al., 2017).

Here we address these gaps in knowledge and evaluate whether there are regional and/or temporal variances in livestock carcass microbiomes. We test and quantify the effect of season (winter and summer) and climatically comparable locations (two UK regions separated by 400 km in an East-West orientation) on commercial broiler poultry carcass bacterial microbiomes from the same breed (Ross 308) reared on the same feed formulation. We go on to quantify whether any environmentally derived microbiome differences persist through the food chain to final retail products.

## 2 Materials and methods

### 2.1 Sample collection

Twenty-eight Ross (Aviagen) 308 breed poultry flocks that were all reared on the same feed formulation were sampled at multiple stages during processing across winter (January–March) and summer (May–August) of 2017. Flocks were equally split between two East-UK and West-UK areas separated by 400 km on a 53–55°N latitude (the precise locations are commercially confidential): these regions are geographically separated but climatically comparable as they are at approximately the same latitude with a mean difference of less than 1 °C and 2 mm rainfall annually across the last decade (Met Office, 2020). Each flock was sampled at three different stages of processing: start – immediately after plucking; mid – after evisceration and a cold carcass rinse; and end – after 48 h in cold storage in retail packaging. Four replicates of five homogenised whole neck flaps were taken aseptically at each sample point. Neck flaps were chosen as they are the accepted area for sampling for *Campylobacter* both within the UK and EU (EU Reg, 2073/2005); samples were placed in a sterile recovery diluent and homogenised using a standard “Stomacher” paddle blender. A total of 336 samples were obtained and immediately transported to the University of Lincoln on dry ice.

### 2.2 DNA extraction and sequencing

DNA was extracted using QIAGEN DNeasy Blood and Tissue DNA extraction kit (QIAGEN GmbH, Hilden, Germany), following the manufacturer's instructions. Samples were fully homogenised and DNA was extracted from a 10 mL sub-sample that was centrifuged at 14,500 rpm and the pellet resuspended in 200 μL of Lysis buffer. Extracted DNA was quantified fluorimetrically using the Quant-iT™ ds DNA assay kit (Tecan Microplate reader, 480 nm, Tecan, Durham, NC, USA) and quality was assessed using NanoDrop™ 2000c Spectrophotometer (Thermo Scientific, Wilmington, DE, USA). The V3/V4 area of the bacterial 16S rRNA gene was PCR-amplified in 50 μL reactions with Kapa HiFi Hotstart Readymix (Kapa Biosystems Inc., Wilmington, MA, USA) using Illumina_16S_341F and Illumina_16S_805R primers (Zheng et al., 2015). PCR products were purified using Agencourt AMPure XP beads according to the manufacturer's instructions (Beckman Coulter, Fullerton, CA, USA). Purified PCR products were sent to the Earlham Institute (Norwich, UK) for library construction and sequencing on an Illumina MiSeq sequencing platform with a 250 bp paired-end read metric. Raw sequences were uploaded on SRA with the following BioProject accession number PRJNA613256.

### 2.3 Bioinformatic analysis

Paired-end 250 bp reads were quality checked using FastQC v0.11.6 (Andrews et al., 2015). QIIME2 (v2018.6) was used to process and analyse the sequence data (Bolyen et al., 2018). Paired end sequences were denoised with ‘dada2’ (Callahan et al., 2016), and clustered into amplicon sequences variants (ASVs) using ‘vsearch’, and then further clustered into ASVs groups with an identity of >97 % (Rognes et al., 2016). Variance-stabilizing normalization (Muletz Wolz et al., 2018) on sequence counts was performed in R v3.6.3 (R Core Team, 2020) using CSS normalization using ‘metagenomeSeq’ and ‘phyloseq’ package (McMurdie and Holmes, 2013; Paulson et al., 2013; Weiss et al., 2017). Bacterial ASVs were annotated using q2-feature-classifier in QIIME2 (v2018.6; Bolyen et al., 2018; Bokulich et al., 2018).

### 2.4 Statistical analysis

The >97 % ASV table was read into R v3.6.3 (R Core Team, 2020) for statistical investigation with the ‘vegan’ package (Dixon, 2003), including the generation of NMDS plots. Compositional dissimilarities among replicates were evaluated through PERMANOVA based on Jaccard (1912) distance metrics based on presence/absence and abundances. Jaccard distances were calculated using the ‘vegdist’ function and PERMANOVA was performed over 1000 permutations using the ‘adonis’ function. ASV numbers (richness) were calculated for each sample and analysed with Kruskal-Wallis tests, and P values were calculated by comparing each value of *H* to the appropriate χ^2^_[*a*-1]_ distribution, where *a* = number of groups, and epsilon-squared estimates of effect size were calculated with *E*^2^ = *H*/((*n*^2^-1)/(*n*+1)), where *n* = number of observations. To identify ASVs that are indicative of factors of interest (season, region, factory stage), an indicator species analysis was conducted using the ‘IndVal’ function of the ‘labdsv’ package in R (Dufrêne and Legendre, 1997). ASVs were classified as significantly indicative at corrected Benjamini-Hochberg false discovery rate (fdr) < 0.05 (Benjamini and Hochberg, 1995).

## 3 Results

Due to commercial constraints that prevented sampling, or loss of sample integrity in transit, 302 of the potential 336 samples went forward for DNA sequencing, and a total of 8,673,822 16S rRNA sequence reads were obtained with an average of 62,212 ± 2266 (mean ± standard error of the mean) reads per sample. 2875 bacterial amplicon sequence variants (ASVs, which we herein call taxa) with >97% similarity were revealed in the sequence data, and these spanned 40 phyla, 83 classes and 127 orders. Table S1 reports ASV information, including season, location and stage of factory process.

The differential seasonal and regional samples allow the influence of these aspects to be evaluated; the first factory stage sample point (immediately after plucking) estimates the microbiome of the incoming flock, and the mid (after evisceration and a cold carcass rinse) and final retail pack sample points evaluate the effect of factory processing on these microbiomes. The final retail pack sample estimates the microbiomes of poultry products destined for human consumption. We chose to evaluate bacterial communities via DNA sequencing using three standard ecological metrics: 1) differences in the absolute numbers of taxa, also known as richness; 2) differences in the types (presence/absence) of taxa, and; 3) differences in the abundances of taxa or community composition (Morrison-Whittle and Goddard, 2015).

### 3.1 The difference in numbers of taxa (richness) across time, space and factory stage

**Fig. 1:**
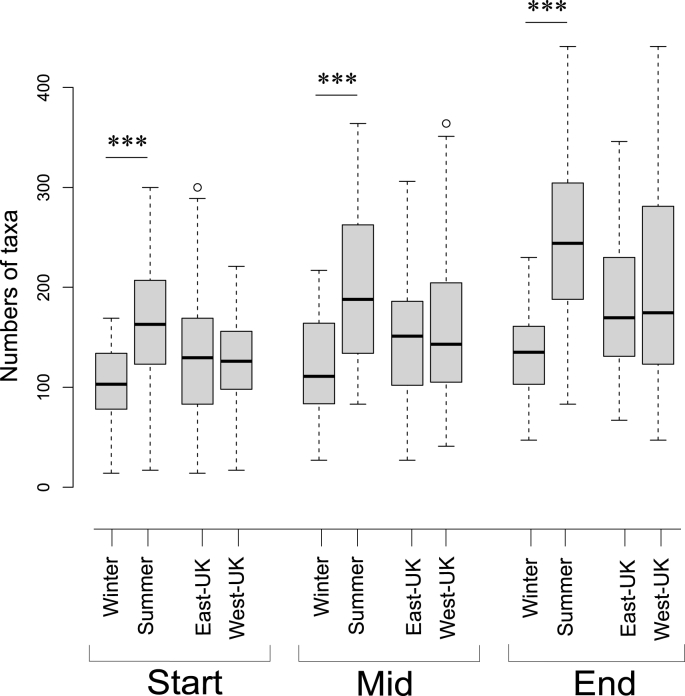
Boxplots of species richness (counts of taxa types) comparing different seasons and UK regions at each stage of the factory (start, middle, end). *** indicates significant differences within factory stages at P < 0.01.

The number of different types bacterial taxa on poultry carcasses was not significantly different between regions (Kruskal-Wallis, P = 0.77, *H* = 0.089; mean 158 and 165 taxa for East-UK and West-UK respectively), but there was a highly significant difference by season (P = 1.2 × 10^−19^, *H* = 82.3), with an average of 84 (or 70%) more taxa per sample in summer than winter (Fig. 1, mean summer and winter 203 and 120 taxa respectively). There was also a highly significant difference in taxa numbers by factory stage (P = 1.5 × 10^−6^, *H* = 23.1): the numbers of taxa increased by an average of ~30 per sample between each stage from 133 taxa at the start to 190 at retail pack (Fig. 1). We compared the relative differences in taxa richness between factory stages in both seasons and regions independently, and the significant difference in taxa number by season, but not region, persisted at each stage in the factory (Table 1). The seasonal difference is greatest on final retail products, with an average of 109 more taxa per sample in summer than winter (Table 1; Fig. 1, summer and winter end 243 and 134 taxa respectively). There was also a significant increase in taxa numbers through the factory in both summer and winter independently. This increase was greatest in summer (P = 1 × 10^−6^, *H* = 23.6), with an average increase of 80 taxa per sample and less but still significant in winter (P = 7 × 10^−4^, *H* = 11.5), with on average increase of 31 taxa per sample.

**Table 1:** Kruskal-Wallis (Numbers), and PermANOVA (Types and Abundances) from 100,000 randomisations, probability results comparing differences by season and region for the numbers, types (via a binary Jaccard dissimilarity matrix) and abundances (via an abundance Jaccard dissimilarity matrix) of taxa at each factory processing stage. Effect sizes (ranging from 0 to 1) are shown as *E*^2^ for Kruskal-Wallis and R^2^ for PermANOVA analyses. Bold indicates strongly statistically significant values (P < 0.001).

	Start		Mid		End	
	P	E^2^/R^2^	P	E^2^/R^2^	P	E^2^/R^2^
**Numbers**						
**Season**	**7x10**^**−7**^	0.250	**6x10**^**−7**^	0.250	**2x10**^**−10**^	0.414
**Region**	0.964	<0.001	0.536	<0.001	0.636	<0.001
**Types**						
**Season**	**9.9x10**^**−5**^	0.0680	**9.9x10**^**−5**^	0.0634	**9.9x10**^**−5**^	0.0791
**Region**	**9.9x10**^**−5**^	0.0620	**9.9x10**^**−5**^	0.0554	**9.9x10**^**−5**^	0.0465
**Abundances**						
**Season**	**9.9x10**^**−5**^	0.0766	**9.9x10**^**−5**^	0.0454	**9.9x10**^**−5**^	0.0486
**Region**	**9.9x10**^**−5**^	0.0634	**9.9x10**^**−5**^	0.0648	**9.9x10**^**−5**^	0.0352

While there are significant differences by both season and factory stage, season explains 3.5-fold more of the variance in taxa numbers than factory stage (*E*^2^ = 0.273 and 0.077 for season and factory stage), showing that season has a relatively larger effect than factory processing on differences in taxa numbers (Fig. 1). Thus, the salient finding is that numbers of taxa on poultry carcasses differ by season, and that seasonal differences are amplified by factory processing. There was a greater increase in taxa numbers from the initial stage to retail products in summer than winter.

### 3.2 The difference in types of taxa across time, space and factory stage

**Fig. 2:**
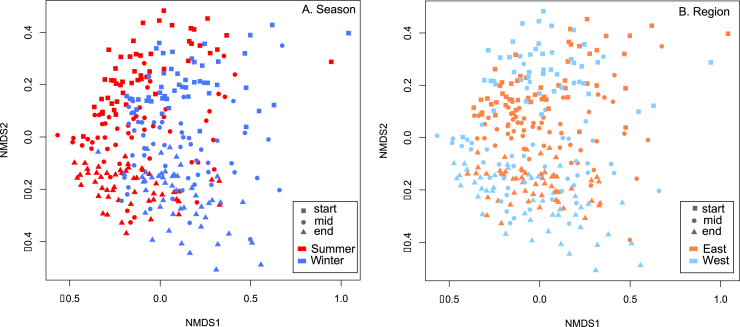
Non-metric Multidimensional Scaling plot reporting pairwise binary Jaccard distances between samples by A) Season and by B) Region, by factory stage.

There was a highly significant difference in the types of bacterial taxa present on poultry carcasses between both seasons and regions (P < 0.0001; Table 1; Fig. 2), with between 4.65 and 7.91% (from the R values) of the variance in taxa presences explained by these factors. There were also highly significant differences in the presences of taxa at different factory stages (P < 0.0001; R^2^ = 0.101; Fig. 2); however, the significant differences by season and region persisted through the factory and remained strongly significant on retail products (Table 1, Fig. 2). We quantified the strength of the effect of season and location on differences in the types of taxa present and compared this to the effect size of transitioning through the factory by comparing the R^2^ values from PERMANOVA analyses, and this revealed that the size of the differences imposed by processing through the factory (~10%) is approximately the same magnitude as imposed by season or region (~8%). The salient finding is that the types of taxa on poultry carcass microbiomes significantly varied by region and season, and these differences were not eroded by factory processing, as the regional and seasonal difference in bacterial taxa types remained strong on retail products.

### 3.3 The difference in abundances of taxa across time, space and factory stage

**Fig. 3:**
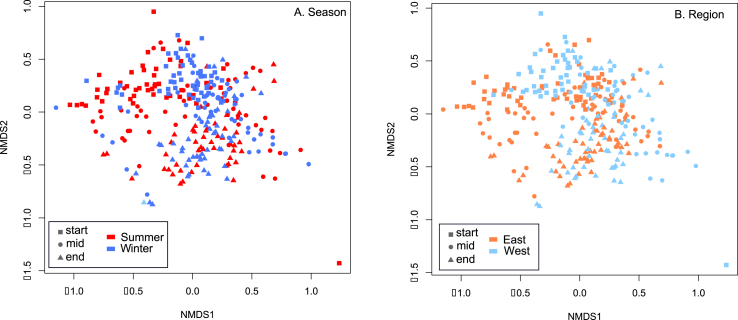
Non-metric Multidimensional Scaling plot reporting pairwise abundance based Jaccard distances between samples by A) Season and by B) Region, by factory stage.

PERMANOVA analyses revealed there were highly significant differences (P < 0.0001; Table 1) in taxa abundances across both seasons and regions that explained 3.53–7.30% of the total variance in bacterial abundances (Table 1; Fig. 3). There were also highly significant differences in the abundances of taxa at different factory stages (P < 0.0001; R^2^ = 0.1109; Fig. 3). The marked difference in abundances between regions and seasons was not eroded by factory processing as highly significant differences in the abundances of bacterial taxa between seasons and regions remained on retail packaged products (Table 1). The size of the effect of factory processing on differences in taxa abundances was slightly greater than that of season and region (R^2^ = 10% and 6% respectively). Again, the salient finding was that abundances of taxa on poultry carcass microbiomes significantly varied by region and season, and these regional and seasonal differences were not diluted by factory processing as the regional and seasonal effect remained strong on retail products (Table 1).

**Fig. 4:**
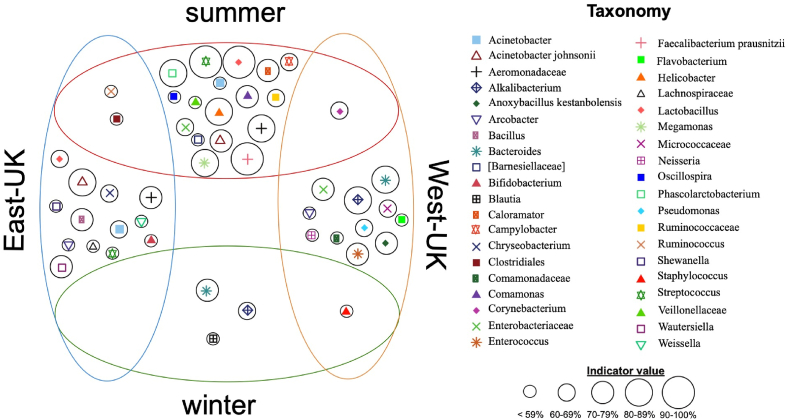
Venn-like diagram showing taxa indicative of seasons and regions. Taxa in the central portions of ellipses are indicative of that region/season generally. Taxa in the peripheral portions of ellipses, that overlap with another ellipsis, are indicative of that region in that season only; e.g. *Campylobacter* are overrepresented in summer across both regions, but *Ruminococcus* is indicative of the East-UK region only in summer. The percent indicator value for each taxa is represented by circle sizes. The genera, or lowest known classification, is shown for each taxa by symbols as described in the figure, data underlying this figure are in Table S2.

Indicator analyses revealed the bacterial genera most over-represented in summer samples (fdr P < 0.05) included *Faecalibacterium, Streptococcus, Lactobacillus, Megamonas*, *Helicobacter*, *Phascolarctobacterium*, and bacterial genera and families most indicative of winter samples included *Bacteroides, Alkalibacterium*, *Staphylococcus, Blautia*, Enterobacteriaceae, Microbacteriaceae, *Arcobacter*, Rikenellaceae, and Micrococcaceae (Fig. 4). *Aeromonadaceae, Acinetobacter, Bacillus*, *Lactobacillus*, *Wautersiella*, *Chryseobacterium*, Clostridiaceae and *Bifidobacterium*, were most indicative of the East-UK samples while *Bacteroides, Alkalibacterium*, *Enterococcus,* Enterobacteriaceae and *Anoxybacillus* are indicative of West-UK ones (Fig. 4). All indicator taxa (fdr P < 0.05) are reported in Fig. 4 and Table S2.

## 4 Discussion

The microbiomes associated with livestock play fundamental roles in animal health and food safety and understanding how both the environment and factory processes interact to influence the types and abundances of microbes associated with products destined for human consumption is a step towards understanding how to best manage these. Here we show that the types and abundances of bacterial taxa on poultry carcasses significantly vary in time and space, and while these also change through factory processing, the seasonal and regional differences remain significant on retail products. Passing through the factory exerts about the same size difference as the ‘environmental’ effects of location and season in terms of defining the types and abundances of microbes associated with poultry carcasses. There were no differences in the absolute numbers of taxa by region, but there were by season, with summer having 70% more taxa types, and the numbers of taxa types on poultry carcasses increased through the factory, especially in summer.

One explanation for the observation of a difference due to factory processes in all aspects of microbiomes, but that transitioning through the factory does not erode the regional and seasonal differences, is that factory processing homogenises microbiomes within a flock as it passes through a factory. Even the largest of sampling efforts will only ever be able to sample a fraction of the 30,000-plus birds in a flock, as our ‘start’ samples did. If there is within flock variance for microbiomes, then samples from birds early in the factory, that have been exposed to fewer of the plant's processing stages, will be less representative of the average microbiome of the entire flock. It is not possible to clean apparatus in-between birds of the same flock meaning microbiomes from flocks become increasingly homogenised by factory processing machinery. Correspondingly, points further down the factory line, where carcasses have been exposed to more machinery, will report microbiomes that are more representative of the homogenised microbiome from the entire flock. If this is the case, the prediction is that samples further down the factory chain will report a greater number of taxa types as these reflect the accumulation of bacteria from the entire flock. This prediction is in line with the observation here that the number of taxa types significantly increased from initial stages to retail packs (Fig. 1). That the signals for region and season are not eroded by this flock homogenisation process signifies the underlying microbiomes associated with flocks differ in space and time.

An average of 6 % of the variance in the types of taxa is explained by region and season (Table 1), meaning the majority of bacterial types are similar between regions and seasons. However, these small differences translate to highly significant differences for the types and abundances of bacterial taxa between regions and seasons. Most of the taxa identified as indicative of a particular season or region are generally also reported from poultry gastrointestinal tracts (Oakley and Kogut, 2016; Sergeant et al., 2014; Wei et al., 2016; Xiao et al., 2017), suggesting that much of this bacterial community come to be on carcasses during processing, as is expected. However, some taxa are not part of expected gut communities, and thus potentially represent bacteria that reside on/in other parts of the birds or are environmentally derived (Fig. 4), and some of these include *Alkalibacterium*, *Jeotgalicoccus*, *Lysinibacillus*, *Akkermansia*, *Christensenellaceae*, *Puniceicoccaceae*, *Fluviicol, Aminiphilaceae* and *Psychrobacter* (also identified from poultry by Handley et al. (2018). *Jeotgalicoccus,* for example, has been isolated from poultry house air (Martin et al., 2010). Some of these taxa have also been reported to be involved in spoilage: *Janthinobacterium lividum* may cause a violet discoloration in rabbit meat (Giaccone et al., 2008). It is possible that variance in carcass associated microbiomes may effect shelf-life or even quality attributes of the final product, as has been shown for horticulture produce (Knight et al., 2015); however, these possibilities remain unexplored for livestock products.

It is important to note that we analysed DNA to evaluate bacterial diversity, and this will have derived from both live and dead bacteria. The diversity of microbial communities associated with retail products provides no measure of food safety generally as certain bacteria such as *Christensenellaceae* (Waters and Ley, 2019)*, Lactobacillus* (Mookiah et al., 2014) and *Bacillus* (Grant et al., 2018), which were recovered here, are correlated with health benefits when consumed by humans. In addition, it is possible more diverse carcass microbiomes may be beneficial as the abundance of benign and beneficial bacteria may prevent human pathogens from becoming established. A minuscule fraction of the microbiome revealed here are negatively associated with food-safety. Just three of the 2875 ASVs and only 0.2% of sequence reads were assigned to the *Campylobacter* genus: the DNA may have derived from dead cells and these bacteria are easily rendered safe with appropriate cooking or freezing. Ninety percent of *Campylobacter* reads were from summer samples, and indicator analysis shows this genus was identified as over-represented in summer (Fig. 4). This is in line with observations of a greater incidence of campylobacteriosis in summer in the UK at least (e.g. Nichols et al., 2012) but to our knowledge this is the first report of differential *Campylobacter* seasonal incidences associated with retail products using NGS approaches. No other human pathogens (*Listeria, Salmonella*) were recovered in the data.

Overall, this is the first objective demonstration of spatial and temporal variance in livestock microbiomes destined for consumption. These flocks had the same genetics (Ross 308) and were produced and managed by the same company including being reared on the same feed formulation from the same supplier. These general spatiotemporal differences correlate with patterns seen in other non-livestock agricultural systems (Bokulich et al., 2014; Taylor et al., 2014). It is tempting to conclude that the differential conditions between regions and seasons contributed to the development of the small but significant differences in poultry microbial communities. However, there are two ecological processes that define why bacterial communities may differ in space and time: natural selection and neutral (chance) processes (Morrison-Whittle and Goddard, 2015). It may well be that differential environmental conditions in these regions and at these times of year subject bacterial communities associated with poultry to different selection pressures, and any difference observed are a result of this. Equally, selection may have had no hand in defining the types and abundances of bacteria: it may just be chance which types of bacteria reside in an area and become associated with any particular flock at a particular time. This experimental design does not allow us to tease these apart; however, differences in mean temperatures in these two regions across the last decade between seasons is marked at ~10 °C (but there is only a ~2 mm difference in rainfall), while overall mean differences between regions are negligible, at <1 °C and 2 mm rainfall (Met Office, 2020).

Lastly, any current and future methods designed to manipulate poultry microbiomes or evaluate the effect of feed (a core component of poultry production) or other husbandry factors need to be cognizant of variation in livestock microbiomes in time and space. For example, a particular microbial control or feed method optimised at one location or in one season may not have the same effect at other locations or times due to differential background bacterial communities. This might be particularly important when designing methods to control human pathogens. Increasing our understanding of livestock microbiomes, the factors that influence them, and how commensals and pathogens interact will help facilitate the implementation of sustainable farming methods that maximise food quality and safety, and possibly reduce antibiotic use in both livestock and humans.

## Declaration of competing interest

The authors declare that they have no competing interests.

## Availability of data and material

The raw amplicon sequencing data from this study was made publicly available in the NCBI BioProject Sequence Read Archive (SRA; https://www.ncbi.nlm.nih.gov/bioproject/) under the BioProject PRJNA613256.
